# Assessment of dental ergonomics knowledge and prevalence of work-related musculoskeletal disorders among dental hygienists in Saudi Arabia: a cross-sectional study

**DOI:** 10.3389/fpubh.2026.1816571

**Published:** 2026-05-29

**Authors:** Rasha N. Alotaibi, Haneen Al-ayyaf, Sarraa Alshibani, Mohammed I. Hashem

**Affiliations:** 1 Dental Health Department, College of Applied Medical Sciences, King Saud University, Riyadh, Saudi Arabia; 2 Dental Hygiene Program, Dental Health Department, College of Applied Medical Sciences, King Saud University, Riyadh, Saudi Arabia

**Keywords:** carpel tunnel syndrome, dental ergonomics, musculoskeletal pain, occupational safety, work-related pain syndrome

## Abstract

Dental professionals are at risk of developing various occupational diseases and disorders, with musculoskeletal disorders (MSDs) being the most prevalent. This study examines the level of knowledge among dental hygienists (students, interns, and licensed practitioners) regarding dental ergonomic practices and investigates the prevalence, common locations, and severity of work-related musculoskeletal disorders (WMSDs). A self-administered and validated bilingual (English/Arabic) questionnaire was constructed utilizing Google Forms and distributed to participants across all regions of Saudi Arabia. The questionnaire included 35 closed-ended items and two open-ended questions, organized into three sections: demographic and professional data, ergonomic practices, and musculoskeletal symptoms. Data was analyzed using SPSS v.22.0. Descriptive statistics summarized categorical variables; chi-square tests enabled comparative analysis. Multivariate logistic regression analysis was also performed to adjust for potential confounding variables. A total of 334 participants completed the survey. Work-related musculoskeletal disorders were reported by 95.3% (*n* = 201) of licensed dental hygienists and 80.5% (*n* = 99) of students, with the neck and lower back identified as the most affected regions. The most frequently reported pain site for both genders was the neck [females 63.4% (*n* = 117); males 64.9% (*n* = 96)], and tension neck syndrome was the most commonly diagnosed condition across the cohort (32.47%). Despite awareness of ergonomic principles, adherence to proper ergonomic practices remains limited. The findings emphasize the need for structured ergonomic education, early preventive interventions, and evidence-based workplace strategies to reduce occupational strain and improve the long-term health and professional sustainability of dental hygienists.

## 1 Introduction

Dental professionals are at risk of developing various occupational diseases and disorders, with musculoskeletal disorders (MSDs) being the most common. MSDs were first documented in 1700 and can lead to long-term health complications (1, 2). The Centers for Disease Control and Prevention define MSDs as “injuries or disorders of the muscles, nerves, tendons, joints, cartilage, and spinal discs” (3). When these disorders are caused by occupational exposure, they are referred to as work-related musculoskeletal disorders (WMSDs). They are notably prevalent among dental professionals, with an average incidence rate of 90.2% (4). The Global Burden of Disease Study also identifies MSDs as the second most common cause of disability worldwide (5). The body regions most frequently impacted by WMSDs among dental professionals include the wrists, elbows, shoulders, neck, and back spine (6). Common symptoms include soreness, numbness, stiffness, puffiness, tenderness, weakness, reduced grip strength, limited range of motion, and loss of coordination (7). Multiple biomechanical and psychosocial risk factors contribute to the development and progression of MSDs. These include repetitive movements, static or improper postures, elevated work demands, insufficient rest breaks, improper equipment adjustments, and inadequate lighting (2, 7–10). Furthermore, an elevated BMI correlates with a greater likelihood of developing musculoskeletal issues, particularly low back pain (11). These findings align with recent evidence indicating a high prevalence of MSDs among healthcare and dental professionals worldwide (12, 13).

A wide range of musculoskeletal conditions such as carpal tunnel syndrome (CTS) (14, 15), sciatica (16, 17), tendinitis (15), tension neck syndrome (TNS) (18), disc herniation (19, 20), trapezius myalgia (21), and rotator cuff tendinitis (15) have been reported among dental professionals including dentists and dental hygienists. Such disorders contribute to loss of working time, decreased performance, the need for medical interventions, and potential early retirement (22, 23). The differences in clinical practice and instrument use between dentists and dental hygienists may influence the prevalence and distribution of WMSDs, particularly in the wrist region. Dental hygienists often rely on ultrasonic devices for scaling, whereas dentists more frequently use high-speed and micromotor handpieces, which involve repetitive and forceful hand movements (24–26).

Musculoskeletal disorders can be mitigated through the application of ergonomic principles and properly designed work environments (21). Ergonomics focuses on optimizing the interaction between individuals and their work environment to enhance safety and performance (27). In the field of dentistry, ergonomic principles, this includes maintaining proper posture, adjusting patient and operator positioning, ensuring appropriate lighting, and using ergonomically designed instruments such as loupes and lightweight handpieces (28, 29). In addition, preventive strategies such as stretching exercises, micro-breaks, and other physical interventions have been suggested to reduce physical strain and improve musculoskeletal health (30–32). These evidence-based ergonomic and rehabilitative approaches have been demonstrated to reduce physical strain and improve musculoskeletal health (26, 30–32). Recent studies have highlighted the continued high prevalence of MSDs among dental professionals and identified gaps in ergonomic practices, particularly in regional settings such as Saudi Arabia (33, 34). However, despite increasing awareness, limited research has specifically focused on dental hygienists, especially in relation to both ergonomic knowledge and the prevalence of WMSDs. Therefore, this study aims to assess the level of ergonomic knowledge among dental hygienists across different experience levels and to evaluate the prevalence, common sites, and severity of WMSDs, as well as the most frequently diagnosed related conditions among dental hygienists in Saudi Arabia.

## 2 Materials and methods

### 2.1 Ethics statement

The study was approved by the Institutional Review Board at King Saud University Medical City (IRB Project No. E-25-9579, dated 12/04/2025). All procedures were conducted in accordance with the ethical standards of the responsible institutional and national research committees, as well as the principles of the 1964 Declaration of Helsinki and its subsequent amendments. Participation in the study was voluntary, and completion of the questionnaire was considered as implied consent.

### 2.2 Sample size

The sample size was calculated using the formula for estimating a population proportion with a specified level of precision (35), According to the Saudi Commission for Health Specialties (SCFHS), the total number of licensed dental hygienists in Saudi Arabia is approximately 1,453. The minimum required sample size was calculated to be 264 based on a confidence level of 90% and a margin of error of 5%. Nevertheless, a total of 334 participants were included in the study to improve its statistical robustness and to account for any potential non-responses.

### 2.3 Study site and target population

A cross-sectional study design was employed using a self-administered questionnaire. Data were collected between January and May 2025 across the Central, Northern, Southern, Western, and Eastern regions of Saudi Arabia. The target population included dental hygiene students (third-year and final-year), internship trainees, and licensed dental hygienists working in clinical or academic settings. Participants were eligible if they were currently engaged in the dental hygiene profession and residing in Saudi Arabia.

### 2.4 Measurement tool

Data were collected using a structured, self-administered questionnaire developed using Google Forms and provided in both English and Arabic to ensure accessibility and clarity for participants across different regions of Saudi Arabia. The questionnaire consisted of three main sections: The first section included demographic characteristics such as age, gender, professional status, and body mass index (BMI). BMI was self-reported and included based on previous evidence suggesting an association between overweight status and an increased risk of musculoskeletal disorders, particularly low back pain. Although self-reported BMI may introduce reporting bias, it is commonly used in epidemiological studies and considered acceptable for large-scale surveys (11). The second section assessed the ergonomic practices and was adapted from a previously validated questionnaire (36). It included items related to dental chair and operator stool adjustment, use of magnification tools (e.g., dental loupes), adequacy of lighting, accessibility and condition of instruments, trunk and neck posture during clinical work, pain associated with vibrating instruments, stretching habits after clinical sessions, and participation in physical activity. As the items were adapted rather than adopted verbatim, minor linguistic modifications were made, and reliability was reassessed accordingly. The third section evaluated MSDs disorders using items adapted from the Standardized Nordic Musculoskeletal Questionnaire (37). It included questions on the presence and location of musculoskeletal pain, symptoms experienced over the past 12 months and the past 7 days, as well as the impact on daily activities, healthcare utilization, sick leave, and treatment approaches such as medication, physiotherapy, or surgery. Pain intensity was measured using the Numeric Pain Rating Scale (0–10), where 0 indicates no pain and 10 represents the worst imaginable pain (38). The survey was distributed electronically via a web link through social media platforms, including WhatsApp and Telegram, to maximize participant reach. The full questionnaire is provided in Appendix A.

### 2.5 Reliability and validity

The questionnaire was validated for face validity through a pilot test with 10 dental hygienists who were not included in the final sample. The pilot test revealed only minor language issues, which were subsequently addressed. Questionnaire reliability was assessed, with Cronbach’s *α* = 0.776, indicating acceptable internal consistency. The questionnaire was also validated by four dental health experts using a 4-point relevance scale (1 = not relevant to 4 = highly relevant) to ensure that the questions were appropriate, relevant, and culturally suitable for dental hygienists in Saudi Arabia. The achieved content validity index (CVI) score of 1.0 demonstrates excellent overall content validity.

### 2.6 Statistical analyses

Statistical analyses were performed using IBM® SPSS® Statistics version 30. Descriptive statistics, including frequencies and percentages, were used to summarize categorical variables such as demographic characteristics, ergonomic practices, pain experiences, and anatomical locations of musculoskeletal symptoms. As the majority of study variables were categorical or ordinal, comparative analyses were conducted using contingency tables. The chi-square test was applied to evaluate associations between the main study groups (dental hygiene students and licensed dental hygienists). When appropriate, Fisher’s exact test was used for variables with small, expected cell counts. These analyses included: (a) comparison of work-related musculoskeletal disorders (WMSDs); (b) comparison of musculoskeletal pain locations; and (c) comparison of categorical pain severity levels between students and licensed dental hygienists. Expected cell counts were assessed to ensure that the assumptions of the chi-square test were met. Corresponding *p*-values were reported to determine statistical significance. All analyses were aligned with the study objectives to compare students and licensed dental hygienists. No parametric tests or normality assessments were performed, as the dataset did not include continuous variables requiring such analyses. Ordinal variables were analyzed using frequency distributions and were included in chi-square comparisons in accordance with standard practices for categorical data analysis. Additionally, multivariate logistic regression analysis was performed to identify factors independently associated with work-related musculoskeletal disorders (WMSDs). The model included professional status, gender, age, and years of clinical experience as independent variables to control for potential confounding effects. Adjusted odds ratios (ORs) with 95% confidence intervals (CIs) were calculated. Model fit was assessed using the Hosmer–Lemeshow goodness-of-fit test. A *p*-value of less than 0.05 was considered statistically significant.

## 3 Results

### 3.1 Sample and regional distribution

A total of 334 participants completed the questionnaire and were included in the analysis. The regional distribution of participants was markedly uneven, with the Central region contributing the vast majority of respondents (84.73%), followed by the South (5.69%), East (3.89%), North (2.99%), and West (2.69%). This regional distribution is consistent with the workforce distribution reported by the SCFHS which indicates a higher numbers of dental hygienists in the Central region. The detailed regional distribution of dental hygienists is presented in Table 1.

**Table 1:** Demographic data among dental hygienists participants.

Parameters	Variables	*N* (%)
Region	Central	283 (84.7%)
	South	19 (5.6%)
	East	13 (3.8%)
	North	10 (2.9%)
	West	9 (2.6%)
Gender	Male	148 (44.3%)
	Female	186 (55.7%)
Age range	19–24 years	122 (36.5%)
	25–35 years	154 (46.1%)
	36–45 years	56 (16.7%)
	46–56 years	2 (0.5%)
Professional status	3rd and 4th year students	90 (26.9%)
	Internee	31 (9.2%)
	Employed (clinical field)	194 (58.0%)
	Employed (academic field)	6 (1.8%)
	Other	13 (3.8%)
Work sector	Governmental sector	65 (30.5%)
	Private sector	130 (61.0%)
	Both sectors	18 (8.4%)
Years of practice	Less than 5 years	83 (38.9%)
	5–10 years	79 (37.%)
	11–20 years	40 (18.77%)
	21–30 years	8 (3.7%)
	31–40 years	3 (1.4%)
Work hours	2–4 h	37 (11.0%)
	6–8 h	65 (19.4%)
	15–30 h	83 (24.8%)
	31–40 h	53 (15.8%)
	>40 h	96 (28.7%)
Dominant hand	Right-handed	300 (89.8%)
	Left-handed	25 (7.4%)
	Both hands equally	9 (2.6%)
BMI score	Below 18.5	12 (3.6%)
	18.5–24.9	152 (45.7%)
	25–29.9	41 (12.3%)
	Higher than 30	10 (3.0%)
	I do not know	117 (35.2%)

### 3.2 Demographics of participants

The sample comprised 186 females (*n* = 186; 55.7%) and 148 males (*n* = 148; 44.3%). Most participants were aged 25–35 years (*n* = 154; 46.1%), followed by those aged 19–24 years (*n* = 122; 36.5%). Regarding professional status, 27.0% were dental hygiene students, 9.9% were interns, 58.0% were clinical dental hygienists, and 1.8% held academic positions. The majority of participants were employed in the private sector (*n* = 130; 61.0%), and 39.0% (*n* = 83) reported having less than 5 years of professional experience. Among the participants, the right hand was the predominant one (*n* = 300; 89.8%). Detailed demographic characteristics are presented in Table 1.

### 3.3 Prevalence of WMSDs

The overall prevalence of self-reported WMSDs was notably high across the cohort. Among licensed hygienists, 95.3% (*n* = 201) reported experiencing WMSD symptoms, compared to 80.5% (*n* = 99) of dental hygiene students. This difference was statistically significant (*χ*^2^ = 16.96, *p* < 0.001) (Table 2).

**Table 2:** Comparison of MSDs and musculoskeletal pain locations among dental hygiene students and licensed dental hygienists.

MSDs and pain locations	Yes/No	Dental hygiene students *n* (%)	Licensed dental hygienists *n* (%)	*χ* ^2^	*p*
WMSDs	Yes	99 (80.5%)	201 (95.3%)	16.96	*p* < 0.0001
	No	24 (19.5%)	10 (4.7%)		
Neck pain	Yes	69 (56.1%)	145 (68.7%)	4.84	0.027^*^
	No	54 (43.9%)	66 (31.2%)		
Both shoulders pain	Yes	50 (40.6%)	110 (52.0%)	3.66	0.056
	No	73 (59.3%)	101 (47.8%)		
Upper back pain	Yes	19 (15.45%)	27 (12.8%)	0.26	0.608
	No	104 (84.5%)	184 (87.2%)		
Middle back pain	Yes	13 (10.6%)	31 (14.6%)	0.82	0.364
	No	110 (89.4%)	180 (85.3%)		
Lower back pain	Yes	54 (43.9%)	121 (57.3%)	5.10	0.024^*^
	No	69 (56.1%)	90 (42.6%)		
Both elbows pain	Yes	8 (6.5%)	19 (9.0%)	0.36	0.548
	No	115 (93.5%)	192 (91.0%)		
Both wrists/hands pain	Yes	26 (21.1%)	47 (22.2%)	0.011	0.916
	No	97 (78.8%)	164 (77.7%)		
Total		123 (36.8%)	211 (63.1%)		

*χ*^2^: Chi-square test score. ^*^p < 0.05 considered statistically significant.

### 3.4 Pain location and group comparisons

The neck and lower back were the most frequently reported pain sites across the sample. Neck pain was reported by 56.1% of students and 68.7% of licensed hygienists, with a statistically significant difference between groups (*χ*^2^ = 4.84, *p* = 0.027). Lower back pain was reported by 43.9% of students compared to 57.4% of licensed hygienists, also showing a statistically significant difference (*χ*^2^ = 5.10, *p* = 0.024). Shoulder pain was reported by 40.7% of students and 52% of licensed hygienists; however, the difference was not statistically significant (*χ*^2^ = 3.65, *p* = 0.055). No statistically significant differences were observed for other anatomical regions, including the upper back, middle back, elbows, and wrists/hands (all *p* > 0.05). Group comparisons were conducted using chi-square tests for categorical variables (presence or absence of pain by body region) (Table 2).

### 3.5 Pain distribution by gender

**Figure 1:**
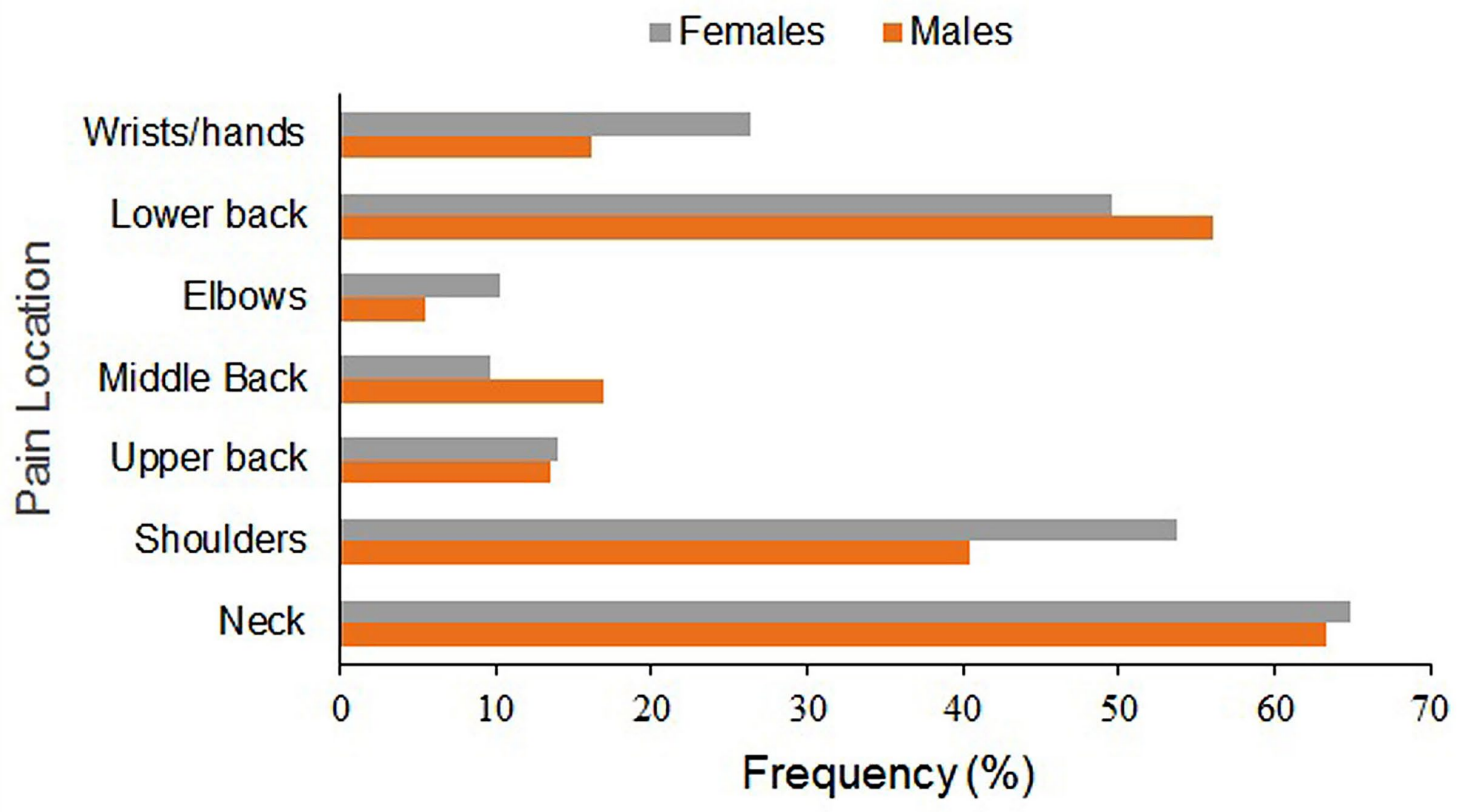
Frequency of musculoskeletal pain location among the participants.

When stratified by gender, the neck was the most frequently reported pain site among both females (63.4%) and males (64.9%). The lower back was the second most common site (females 49.5%; males 56.1%), followed by the shoulders, wrists/hands, upper back, and middle back (Figure 1). Chi-square analysis comparing males and females (presence or absence of pain by body region) showed no statistically significant differences across most anatomical sites (all *p* > 0.05), except for the wrists/hands. Wrist/hand pain was reported by 26.3% of females and 16.2% of males, with a statistically significant difference between groups (*χ*^2^ = 4.37, *p* = 0.036).

### 3.6 Pain severity

**Figure 2:**
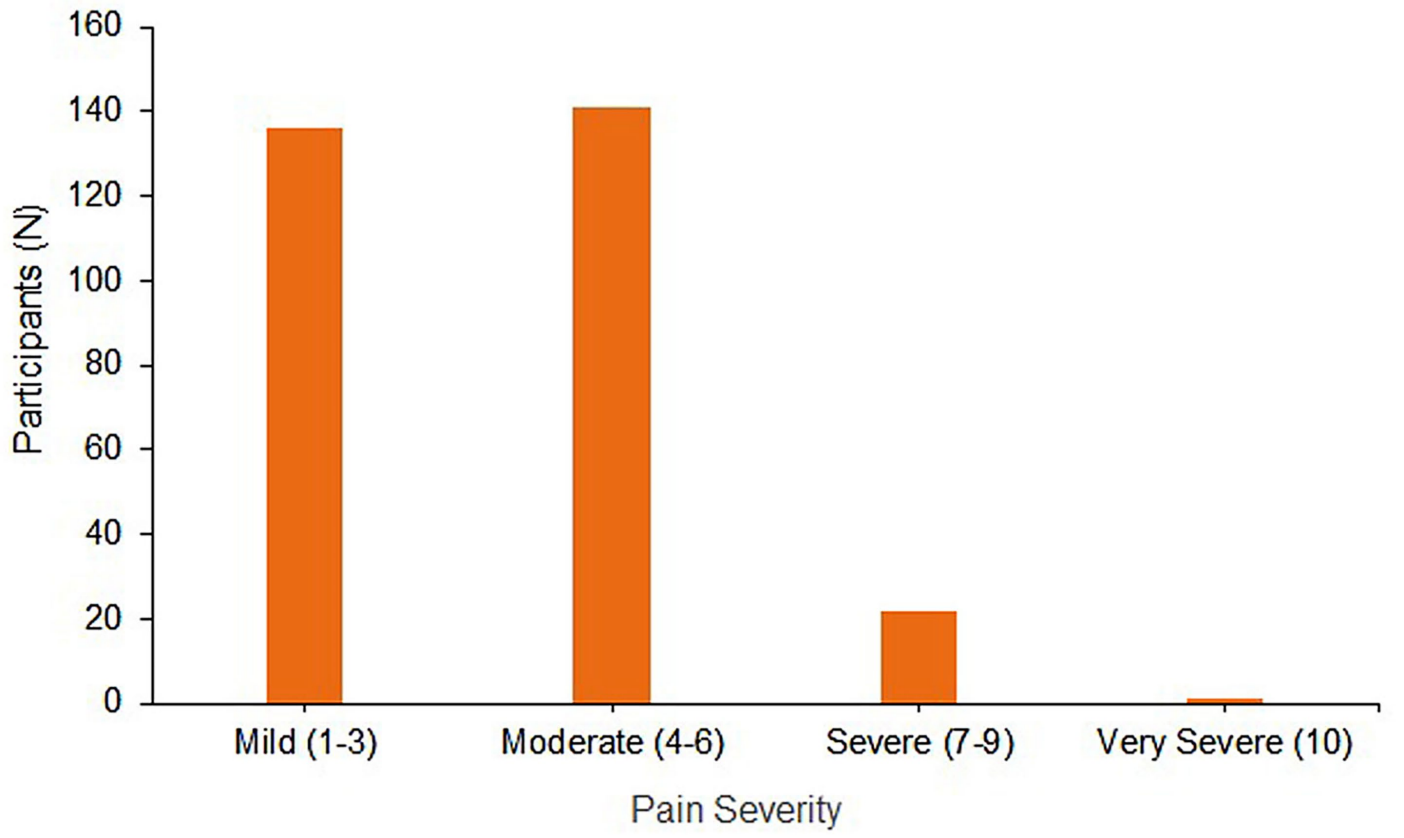
Severity of pain among the participants (numeric scale).

Pain severity was assessed using a numerical rating scale (0–10) and categorized as mild (1–3), moderate (4–6), severe (7–9), and very severe (10) (Figure 2). Across the cohort, moderate pain was the most frequently reported category (47.0%, *n* = 141), followed by mild pain (45.3%, *n* = 136). Severe pain was reported by 7.3% (*n* = 22) of participants, while very severe pain was reported by 0.3% (*n* = 1). When comparing groups, a significantly higher proportion of licensed dental hygienists reported moderate pain compared to students (53.7% vs. 33.3%; *χ*^2^ = 4.664, *p* < 0.001). No statistically significant differences were observed between groups for mild, severe, or very severe pain categories.

### 3.7 Clinically diagnosed musculoskeletal disorders

**Figure 3:**
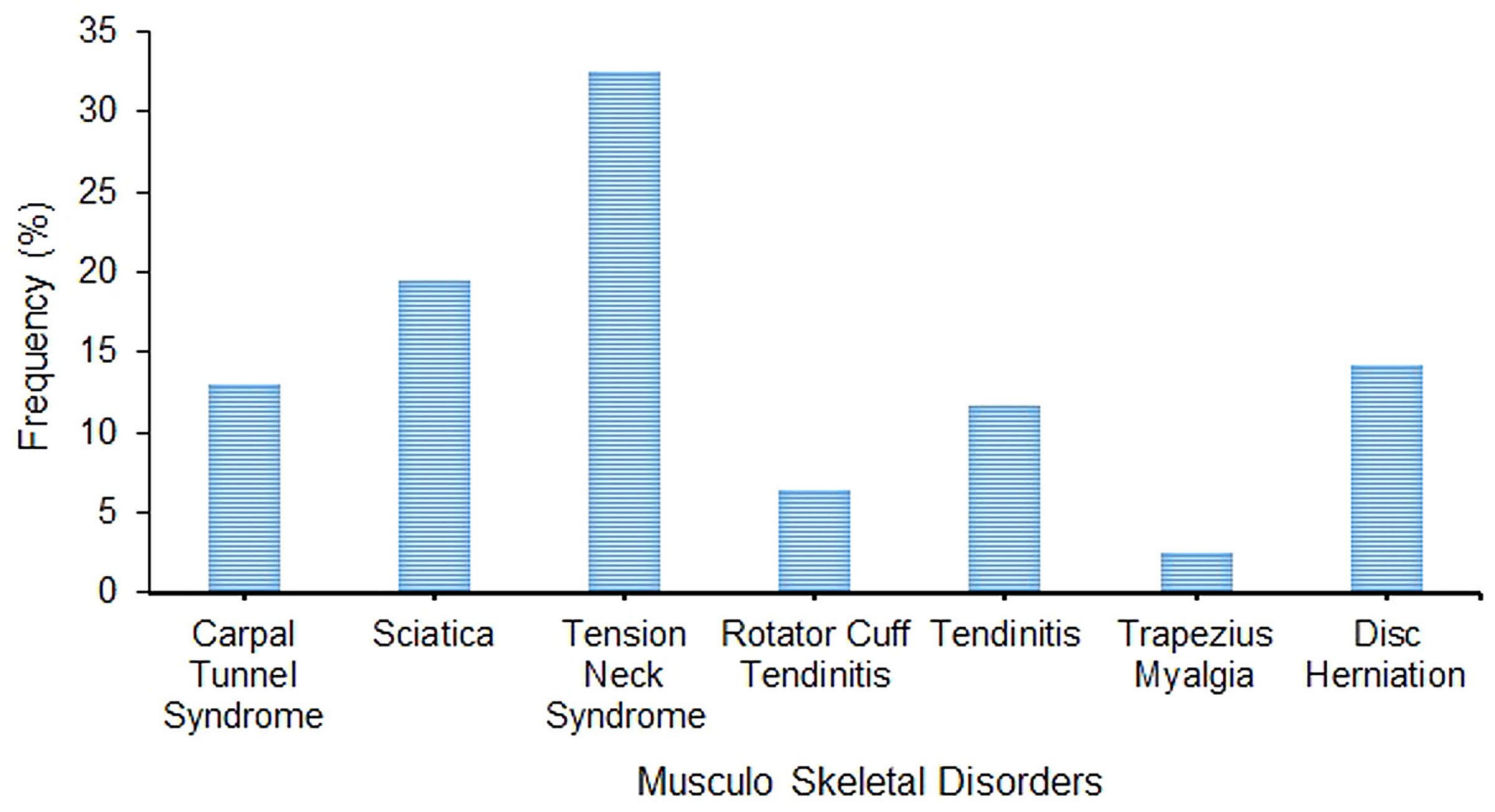
Frequency of diagnosed MSDs among the participants.

The distribution of diagnosed conditions is presented in Figure 3. The participants were asked whether they had received a clinical diagnosis for specific MSDs. Among licensed dental hygienists, the most frequently diagnosed conditions were TNS (9.95%), sciatica (6.79%), and disc herniation (4.98%). Tendinitis and CTS were reported less frequently as diagnosed conditions (Tendinitis 2.71%; CTS 3.62%). Among students, diagnosed conditions were less common, with TNS and tendinitis each reported by 3.2% of participants, while other diagnoses were rare or not reported. A significantly higher proportion of students reported no clinical diagnosis compared with licensed dental hygienists (91.5% vs. 68.8%).

### 3.8 Pain relief interventions and health-care utilization

**Figure 4:**
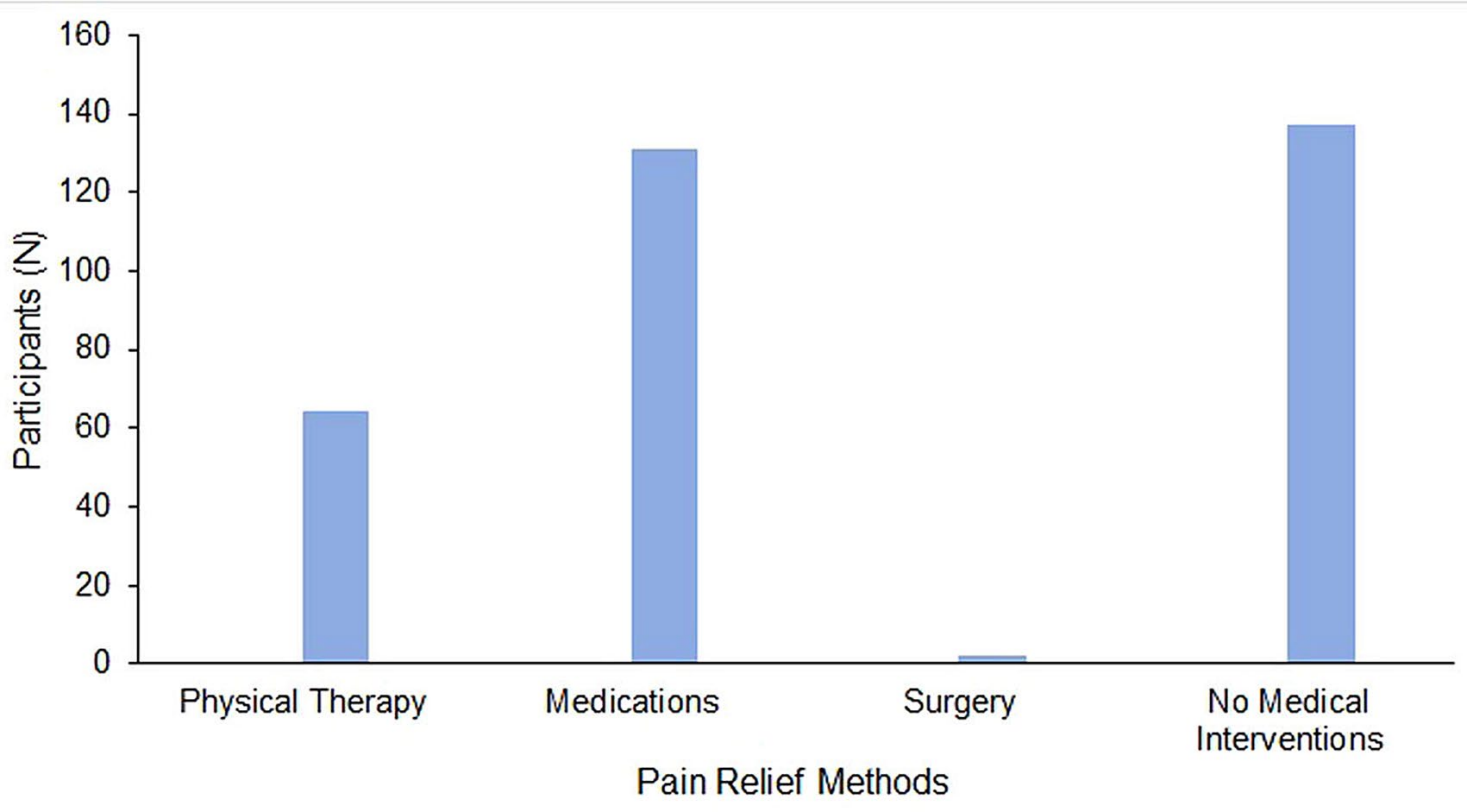
Distribution of pain relief methods among the participants.

Regarding pain management strategies, medication was the most commonly reported intervention (39.3%), followed by physical therapy (19.1%). A total of 41.0% of participants reported not seeking any treatment, while surgical intervention was rare (0.6%) (Figure 4). Regarding pain burden, 74.3% reported musculoskeletal pain within the past 12 months, and 44.3% reported pain in the past 7 days. Activity limitation due to pain over the past 12 months was reported by 20.7%, while 25.3% reported seeking medical consultation. Additionally, 16.3% reported taking sick leave due to pain.

### 3.9 Ergonomic practices

Self-reported ergonomic practices are summarized in Table 3. Most participants reported awareness of proper ergonomic posture (92.5%) and having instruments within easy reach (96.4%). The majority (75.4%) reported adjusting the dental chair or stool to maintain proper posture, whereas only 14.1% reported regular use of dental loupes or magnification devices. Stretching exercises after clinical work were reported by 9.0% of participants, while 22.1% engaged in regular physical activity. Approximately 13.5% reported pain associated with the use of vibrating instruments. Overall, these findings indicate a gap between ergonomic awareness and the consistent implementation of preventive practices.

**Table 3:** Frequency of ergonomic practices among dental hygienist participants.

Ergonomic practices and exercising	Response	*N* (%)
Familiarity with good ergonomic posture in the clinic	Yes	309 (92.0%)
	No	25 (7.5%)
Adjusting the dental chair and stool for good ergonomic posture	Yes	252 (75.4%)
	No	82 (24.6%)
Using dental loupes or magnifying glasses at work	Yes	47 (14.0%)
	No	287 (85.9%)
Help of loupes or magnifying glasses with posture	Yes	36 (76.6%)
	No	11 (23.4%)
Instruments are within hand’s reach without difficulty	Yes	322 (96.4%)
	No	12 (3.6%)
Experiencing pain when using vibrating instruments	Yes	45 (13.5%)
	No	289 (86.5%)
Performing torsions/cervical flexions for better vision while working	Yes	162 (48.5%)
	No	172 (51.5%)
Ensuring sufficient light is available in the workplace	Yes	196 (58.7%)
	No	138 (41.3%)
Instruments in optimal conditions without the need for extra work	Yes	175 (52.4%)
	No	159 (47.6%)
Performing stretching exercises after clinical practice	Yes	30 (9.0%)
	No	304 (91.0%)
Engaging in any sports or exercises	Yes	73 (22.1%)
	No	261 (77.9%)

### 3.10 Multivariate analysis

A multivariate logistic regression analysis was conducted to assess factors associated with WMSDs after adjusting for potential confounders. The overall model was statistically significant (*χ*^2^ = 14.17, *p* = 0.007), indicating that the included variables contributed to the prediction of WMSDs. Professional status was significantly associated with WMSDs, with licensed dental hygienists demonstrating higher odds of reporting musculoskeletal disorders compared with students (*p* = 0.020). Gender was also identified as a significant predictor (*p* = 0.034). In contrast, age (*p* = 0.749) and years of clinical experience (*p* = 0.449) were not significantly associated with WMSDs in the adjusted model. The Hosmer–Lemeshow test indicated good model fit (*p* = 0.951) (Table 4).

**Table 4:** Multivariate logistic regression analysis of factors associated with work-related musculoskeletal disorders (WMSDs).

Variable	*B*	S.E.	*p*-value	OR (Exp B)	95% CI (Lower–Upper)
Professional status	1.086	0.466	0.020	2.964	1.188–7.391
Gender	−0.568	0.268	0.034	0.566	0.335–0.958
Age	−0.114	0.355	0.749	0.892	0.445–1.791
Years of experience	−0.170	0.224	0.449	0.844	0.544–1.310

^*^p < 0.05 considered statistically significant.

*B*, regression coefficient; S.E., standard error; OR, odds ratio; CI, confidence interval.

The model was adjusted for professional status, gender, age, and years of experience.

Model statistics: *χ*^2^ = 14.17, *p* = 0.007. Hosmer–Lemeshow test: *p* = 0.951.

## 4 Discussion

### 4.1 Overview of WMSDs prevalence between licensed dental hygienists and students

Previous studies have consistently reported a high prevalence of WMSDs among dental professionals, with rates ranging from 64 to 93% (9). These findings highlight the substantial ergonomic demands inherent in dental work. Similarly, the current study demonstrated a high prevalence of WMSDs among dental hygienists in Saudi Arabia, with 95.3% of licensed professionals and 80.5% of students reporting musculoskeletal symptoms. This indicates that professional practice is associated with a greater musculoskeletal burden. The higher prevalence observed among licensed dental hygienists may be attributed to prolonged working hours, increased weekly workload, and cumulative years of clinical exposure. In contrast, students typically engage in shorter clinical sessions with more structured breaks, potentially reducing musculoskeletal strain. This exposure-dependent pattern supports the inclusion of both groups, as it enables the identification of early symptoms during training and highlights the progression of WMSDs with continued occupational exposure. These findings are consistent with previous literature (22) which reported higher prevalence rates among practicing hygienists compared to students Neck pain was significantly more prevalent among licensed dental hygienists (68.7%) compared with students (56.1%), which aligns with the higher frequency of TNS reported among professionals (10.0%) compared with students (3.2%). This may be related to prolonged static postures, sustained cervical flexion, and repetitive clinical tasks. A previous study has also reported a high prevalence of neck pain among dental hygiene students, suggesting that symptoms may begin early and progress with increased clinical workload (18). Moreover, a recent study (11) conducted among dental students in Saudi Arabia reported a high prevalence of WMSDs, reinforcing the importance of early assessment and preventive interventions during academic training to reduce long-term occupational consequences. Collectively, these findings emphasize the importance of early ergonomic training and sustained preventive strategies.

### 4.2 Gender-based differences

Previous research has demonstrated that female dental professionals experience higher rates of WMSDs compared to males (39–41), which may be related to physiological and occupational factors such as muscle strength differences and hormonal influences (42, 43). Similarly, the findings of this study showed that female participants reported higher levels of musculoskeletal pain, supporting existing evidence on gender-related differences in WMSD risk.

### 4.3 Workplace sector

Previous studies have suggested that dental professionals working in private sector settings may experience higher musculoskeletal strain due to increased patient load, longer working hours, and limited ergonomic regulations (41). Consistent with these findings, the present study demonstrated a significantly higher prevalence of WMSDs among licensed hygienists (95.4%) compared with students (80.2%) (*χ*^2^ = 16.93, *p* = 0.000). This may indicate that private sector practice environments contribute to increased ergonomic demands and musculoskeletal burden.

### 4.4 Pain distribution and diagnosed disorders

Previous research has identified BMI as a potential risk factor for musculoskeletal disorders (44). However, the current study did not find a statistically significant association between BMI and WMSDs, which may be related to the relatively homogeneous BMI distribution among participants.

In contrast to international findings that report higher prevalence of wrist pain among dental hygienists, the present study found that neck pain was the most commonly reported symptom among both females (63.4%) and males (64.9%), followed by low back pain. This difference may be related to ergonomic practices, particularly the low use of dental loupes (14.1%), which may increase cervical strain due to forward head posture. Although carpal tunnel syndrome is frequently reported in the literature (45). The current findings suggest a lower prevalence of wrist-related symptoms. This may be explained by the greater reliance on ultrasonic instruments, which require less force compared to manual instrumentation. In this study, 86.5% of participants reported no pain when using vibrating instruments, supporting this interpretation. Notably, TNS was the most commonly reported diagnosis, which may reflect differences in ergonomic behavior, workload, and preventive practices. These findings highlight the importance of context-specific ergonomic assessments.

### 4.5 Ergonomic practices and pain relief

Previous studies have identified stretching exercises as a common method for managing musculoskeletal discomfort (11). However, the present study found that medication was the most frequently used method among dental hygienists (39.3%), followed by physical therapy, while only a small proportion reported practicing stretching exercises. This may suggest a preference for immediate symptom relief over preventive strategies in clinical practice.

### 4.6 Strengths and limitations

A major strength of this study is the relatively large sample size and the inclusion of both dental hygiene students and licensed dental hygienists, which enabled meaningful comparisons across different levels of clinical experience. The use of previously validated instruments, including items adapted from the Nordic Musculoskeletal Questionnaire and an ergonomic practices scale, enhances the methodological rigor of the study. The internal consistency of the multi-item sections was acceptable (Cronbach’s *α* = 0.776), indicating satisfactory reliability.

However, several limitations should be acknowledged. The cross-sectional design limits the ability to infer causal relationships between ergonomic practices and musculoskeletal disorders. In addition, reliance on self-reported data may introduce recall and reporting bias. The regional distribution of participants was skewed toward the central region, which may limit the generalizability of the findings to the broader population of dental hygienists in Saudi Arabia. Furthermore, the use of self-reported BMI and self-reported clinical diagnoses may reduce the accuracy of these variables.

### 4.7 Recommendations for future research

Future studies should adopt longitudinal designs to better assess temporal changes in ergonomic behaviors and musculoskeletal symptoms. Intervention-based research is also recommended to evaluate the effectiveness of ergonomic training programs, stretching exercises, loupe utilization training, and workplace modifications in reducing musculoskeletal risk. In addition, the use of objective assessment methods, such as posture analysis systems and electromyographic muscle activity measurements, may provide more accurate insights into ergonomic risk factors.

## 5 Conclusion

This study demonstrates a high prevalence of musculoskeletal disorders among dental hygienists and students in Saudi Arabia, highlighting a significant occupational health concern. Although a moderate level of awareness regarding ergonomic principles was observed, inconsistent application of these practices may contribute to the high burden of musculoskeletal symptoms. The findings emphasize the need for structured ergonomic education, early preventive interventions, and evidence-based workplace strategies to reduce occupational strain and improve the long-term health and professional sustainability of dental hygienists.

## Data Availability

The original contributions presented in the study are included in the article/Supplementary material, further inquiries can be directed to the corresponding author.
