# Supplementary material for: Assessment of dental ergonomics knowledge and prevalence of work-related musculoskeletal disorders among dental hygienists in Saudi Arabia: a cross-sectional study
Source: Front Public Health. 2026 May 29;14:1816571. doi: 10.3389/fpubh.2026.1816571 (PMC13259716; doi:10.3389/fpubh.2026.1816571)
Supplement: Supplementary file 1 [file Data_Sheet_1.pdf]

Appendix A

Study Questionnaire

Section I: Demographic and dental practice characteristics

Q1. Are you a dental hygienist?
a) Yes
b) No

Q2. Do you live in Riyadh?
a) Yes
b) No

Q3. If you answered "No" to the previous question, in which region of the Kingdom of Saudi Arabia do you live?
a) Central Region
b) Northern Region
c) Southern Region
d) Western Region
e) Eastern Region

Q4. What is your gender?
a) Male
b) Female

Q5. What is your age range?
a) 19-24 years
b) 25-35 years
c) 36-45 years
d) 46-56 years
e) >56 years and above

Q6. Select the category that best describes your current professional status as a dental hygienist.
a) Undergraduate dental hygiene student (studying and yet to graduate).
b) Internship dental hygiene student (graduated and still completing internship training).
c) Employed dental hygienist (working in the field as a professional).
d) Employed dental hygienist (Working in the academic field).

Q7. If you are an undergraduate student, which year are you in?
a) First year of the program (level 3-4)
b) Second year of the program (level 5-6)
c) Third year of the program (level 7-8)

Q8. If you are a student, an intern, or employed, how many hours do you work per week? -2-4 hrs/ week
a) 6-8 hrs / week
b) 15-30 hrs/ week
c) 31-40 hrs/ week
d) >40 hrs/week

Q9. If you are employed, in which sector do you work?

a) Governmental sector
b) Private sector
c) Both

Q10. If you are employed, how many years have you been practicing as a dental hygienist?

a) Less than 5 years
b) 5-10 years
c) 11-20 years
d) 21-30 years
e) 31-40 years
f) More than 40 years

Q11. As a working dental hygienist, what is your dominant hand?

a) Right-handed
b) Left-handed
c) I use both hands equally

Q12. What is your weight?

a) 40-50 kg
b) 51-60 kg
c) 61-70 kg
d) 71-80 kg
e) 81-90 kg
f) above 90 kg

Q13. What is your height?

a) 140-150 cm
b) 151-160 cm
c) 161-170 cm
d) 171-180 cm
e) 181-190 cm
f) > 190 cm

Q14. What is your BMI score?

a) below 18,5 underweight
b) 18,5 to 24,9 normal
c) 25 to 29,9 overweight
d) Higher than 30
e) I do not know

Ergonomic practices and exercising

Q15. Are you familiar with good ergonomic posture practices in the clinic?

a) Yes
b) No
c) I am not familiar with good ergonomic posture practices in the clinic

Q16. Do you adjust the dental chair for the patient and the dental stool for yourself for good ergonomic posture?
a) Yes
b) No
c) Sometimes

Q17. Do you use dental loupes or magnifying glasses at work?
a) Yes
b) No
c) Sometimes

Q18. If you answered yes or sometimes to the previous question, did it help with your posture during work?
a) Yes
b) No
c) It did not help that much

Q19. Are your instruments within hand's reach without difficulty?
a) Yes
b) No

Q20. Do you experience pain when using vibrating instruments (ultrasonic dental scaler, handpiece)?
a) Yes
b) No
c) Sometimes

Q21. Do you perform torsions or cervical flexions for better vision while working? -Yes
a) No
b) Sometimes

Q22. Do you ensure sufficient light is available in the workplace?
a) Yes
b) No
c) Sometimes

Q23. Are the instruments in optimal conditions (lightweight and balanced) without the need to do extra work (i.e sharped working instruments)?
a) Yes
b) No
c) Sometimes

Q24. Do you perform stretching exercises after clinical practice?
a) Yes, regularly
b) Yes, sometimes
c) No, never

Q25. Do you engage in any sports or exercises?

a) Yes, regularly
b) Yes, sometimes
c) No, never

Section III: Musculoskeletal and Severity of Pain

Q26. Have you experienced musculoskeletal pain due to work as a dental hygienist?

a) Yes, consistently
b) Yes, sometimes
c) No, never

Q27. How severe is the pain on a scale of 0-10 (Numeric Scale)?

a) 0 no pain
b) 1-3 mild
c) 4-6 moderate
d) 7-9 severe
e) 10 very severe

Q28. If yes, where is the pain located?

a) Neck
b) Both shoulders
c) Left shoulder
d) Right shoulder
e) Upper back
f) Lower back
g) Both elbows
h) Left elbow
i) Right elbow
j) Both wrists/hands
k) Left wrists/hand
l) Right wrists/hand
m) Both hips/thighs
n) Left hip/thigh
o) Right hip/thigh
p) Both knees
q) Left knee
r) Right knee
s) Both ankles/feet
t) Left ankle/foot
u) Right ankle/foot

Q29.Have you at any time during the last 12 months had trouble (ache, pain, discomfort) in [the selected body region that you select in question 28]?

a) Yes
b) No

Q30. Have you at any time during the last 7 days had trouble (ache, pain, discomfort) in [the selected body region that you select in question 28]?

a) Yes
b) No

Q31. During the last 12 months, have you been prevented from carrying out normal activities (e.g., job, housework, hobbies) because of this trouble?

a) Yes
b) No

Q32. During the last 12 months, have you seen a physician or other healthcare professional for this condition?

a) Yes
b) No

Q33. Have you at any time during the last 12 months taken sick leave because of this trouble in [the selected body region that you select in question 28?

a) Yes
b) No

Q34. What interventions have you sought for pain relief?

a) Physical Therapy
b) Medications
c) Surgery
d) I have not undergone any medical interventions

Q35. Have you been diagnosed with some of these MSDs?

a) Carpal tunnel syndrome
b) Sciatica
c) Tendinitis
d) Tension neck syndrome
e) Trapezius myalgia
f) Rotator cuff tendinitis
g) Disc herniation
h) I haven't been diagnosed with this MSDs
i) Other
